# Progress and perspective of *TBX6* gene in congenital vertebral malformations

**DOI:** 10.18632/oncotarget.10619

**Published:** 2016-07-15

**Authors:** Weisheng Chen, Jiaqi Liu, Dongtang Yuan, Yuzhi Zuo, Zhenlei Liu, Sen Liu, Qiankun Zhu, Guixing Qiu, Shishu Huang, Philip F. Giampietro, Feng Zhang, Nan Wu, Zhihong Wu

**Affiliations:** ^1^ Department of Orthopaedic Surgery, Peking Union Medical College Hospital, Peking Union Medical College and Chinese Academy of Medical Sciences, Beijing, China; ^2^ Chinese Academy of Medical Sciences and Peking Union Medical College, Beijing, China; ^3^ Beijing Key Laboratory for Genetic Research of Skeletal Deformity, Beijing, China; ^4^ Breast Surgical Oncology, Cancer Hospital of Chinese Academy of Medical Sciences, Beijing, China; ^5^ Department of Orthopaedics, Huai'an First People's Hospital, Nanjing Medical University, Huai'an, Jiangsu, China; ^6^ Department of Orthopaedic Surgery, West China Hospital, Sichuan University, Chengdu, China; ^7^ Department of Pediatrics, University of Wisconsin School of Medicine and Public Health, Madison, WI, USA; ^8^ State Key Laboratory of Genetic Engineering, School of Life Sciences, Fudan University, Shanghai, China; ^9^ Medical Research Center of Orthopaedics, Chinese Academy of Medical Sciences, Beijing, China; ^10^ Department of Central Laboratory, Peking Union Medical College Hospital, Peking Union Medical College and Chinese Academy of Medical Sciences, Beijing, China

**Keywords:** congenital vertebral malformation, TBX6, congenital scoliosis, somitogenesis, vertebrate segmentation

## Abstract

Congenital vertebral malformation is a series of significant health problems affecting a large number of populations. It may present as an isolated condition or as a part of an underlying syndromes occurring with other malformations and/or clinical features. Disruption of the genesis of paraxial mesoderm, somites or axial bones can result in spinal deformity. In the course of somitogenesis, the segmentation clock and the wavefront are the leading factors during the entire process in which *TBX6* gene plays an important role. *TBX6* is a member of the T-box gene family, and its important pathogenicity in spinal deformity has been confirmed. Several *TBX6* gene variants and novel pathogenic mechanisms have been recently revealed, and will likely have significant impact in understanding the genetic basis for CVM. In this review, we describe the role which *TBX6* plays during human spine development including its interaction with other key elements during the process of somitogenesis. We then systematically review the association between *TBX6* gene variants and CVM associated phenotypes, highlighting an important and emerging role for *TBX6* and human malformations.

## INTRODUCTION

Congenital Vertebral Malformations (CVM) represent a group of serious birth defects, which may present as congenital scoliosis, kyphosis, Klippel Feil syndrome (OMIM 118100) [1] (short neck and low posterior hairline in association with cervical vertebral fusion) and may occur in conjunction with other birth defects or as part of an underlying genetic syndrome. The prevalence of CVM is approximately 0.5-1.0 per 1000 persons [2]. Some patients' lack of overt deformity may lead to a delayed or missed diagnosis, thus the actual proportion of CVM may be greater [3]. Other syndromes associated with CVM include: Alagille syndrome (OMIM 118450: intrahepatic cholestatis, pulmonic stenosis, embryotoxon and butterfly vertebral anomaly in association with distinct facial features) [4], spondylocostal dysostosis (OMIM 122600 : contiguous vertebral malformations throughout the spine with a characteristic “pebble beach sign” in conjunction with rib malformations)(SCD) [5, 6], VACTERL syndrome [7] (OMIM 314390: V = vertebral malformations; A = anal abnormalities, C = cardiac malformations; TE = tracheoesophageal fistula and L = Limb malformations), Goldenhar syndrome (OMIM 164210) [8, 9] and spondylothoracic dystrophy (STD-vertebral malformations with ribs emanating from a common point of origin) [10, 11] and others reviewed in.

CVMs occur as a result in altered development of the paraxial mesoderm, somites or axial skeleton [12]. In vertebrate embryogenesis, the paraxial mesoderm lies adjacent to the neural tube and develops from the anterior portion of the embryo into somites during a specific process which is called somitogenesis. This process is mainly regulated by WNT, FGF and Notch signaling pathways. Each somite is later subdivided into the ventromedial sclerotome (which the vertebral body is derived from) and the dorsolateral dermomyotome (from which body skeletal muscles and dorsal dermis arise). Any defect of paraxial mesoderm or somite formation may contribute to CVM [13, 14]. CVM represent a complex condition with multiple causes which remain to be elucidated.

## THE ESSENTIAL ROLE OF TBX6 IN SOMITOGENESIS

*TBX6* gene is known as *T-box 6*, a member of the *T-box* family, and encodes a transcription factor which plays an important role in the regulation of development process [15]. *TBX6* has been localized to 16p11.2, with a 6,095bp in size, and contains 8 exons. *Tbx6*, the *TBX6* homologous gene, is located in the chromosome 7 in mouse genome [15]. In the zebrafish genome, *tbx24* gene in chromosome 12 is suggested to be the homologous gene with mice *Tbx6* and human *TBX6* [16].

*Tbx6* gene codes for a 1.9-kb transcript which could be detected in the embryonic part of the egg cylinder at 7.0 days post coitus (dpc) [17]. The *Tbx6* transcripts are in a stripe which is equivalent to the early primitive streak. At 7.5 dpc, the expression of *Tbx6* was detected in the primitive streak, extending caudally along the allantois from the node to the base as well as in the paraxial mesoderm surrounding the streak. At 8.5 dpc, the expression of *Tbx6* transcripts was identified in the presomitic mesoderm (PSM) of the tail portion (unsegmented), surrounding the caudal end of the neural plate. The expression of *Tbx6* in tailbud was positive until 12.5 dpc and become negative after 13.5 dpc. Thus, the expression of *Tbx6* in the primitive streak, tailbud and PSM has an important influence on the development process. Additionally, *Tbx6* mutation affects the differentiation of paraxial mesoderm [18]. *Tbx6* gene was mutated through homologous recombination in an embryonic stem, which deleted the initiating methionine of *Tbx6* and established the mutant allele *Tbx6^tm1Pa^*. The *Tbx6*/ *Tbx6^tm1Pa^* were viable, and the offspring were detected without obvious abnormalities as well as homozygotes. The histological segmentation abnormalities were detected firstly at embryonic day of development (E)8.5. At E9.5 and E10.5, an enlarged tail bud was observed that was full of large numbers of undifferentiated mesenchymal cells. In the expanded tail bud and abnormal somites, three mesoderm markers, *Delta-like gene 1* (*Dll1*), *paraxis* and *Mox-1* remained negative. These observations supported the important role *Tbx6* plays during somitogenesis.

Sex determining region Y-box 2 *(Sox2)* regulated by *Tbx6* is essential for the specification of paraxial mesoderm from the axial stem cells [19]. The enhancer N1 of *Sox2* is activated in the caudal lateral epiblast (CLE), to maintain the cells in the superficial layer of the neural plate sustaining the activation of N1 and *Sox2* expression. The cells which developed into mesoderm activate *Tbx6* to turn off enhancer N1 before the migration into the paraxial mesoderm. In contrast to this, *Tbx6* mutants display persistence of enhancer N1 activity in the paraxial mesoderm, eliciting ectopic *Sox2* activation which results in transformation of the paraxial mesoderm into neural tube. Introduction of the N1-specific deletion mutation into *Tbx6* mutants prevented the development of neural tube formation due to the prevention of ectopic *Sox2* activation in the mesodermal compartment, indicating that *Tbx6* regulated *Sox2* through enhancer N1. Additionally, *Tbx6*-dependent repression of *Wnt3a* was also involved in this process. After the paraxial mesoderm-specific misexpression of a *Sox2* transgene, ectopic development of neural tubes was formed in the wild type embryos. This is in consistent with the findings that Wnt signaling pathway regulated the differentiation of paraxial mesoderm through the axial stem cells [20].

## MECHANISM OF THE SOMITOGENESIS AND THE CLOCK-WAVEFRONT MODEL

Embryonic development of somite is regulated by a variety of factors. The most widely accepted model of somitogenesis is the clock-wavefront model [21, 22]. Cooke et al [23] proposed the clock and the wavefront model in 1976 for the first time. Pourquie et al [24] further verified the existence of segmentation clock and identified Notch signaling pathway which plays an important role in the formation of somites. Expression of cyclic genes is periodic to form one wave of expression passes through the PSM during the formation of one somite [25-28]. Notch signaling is essential for the separation of intermingled PSM cells belonging to adjacent segments and for intrasomitic anterior-posterior patterning [29-31]. In mice and zebrafish, mutations of Notch pathway genes may lead to the loss of periodic expression patterns. Some mutations in Notch pathway components will lead to defects in somitogenesis, the expression of cyclic genes [27, 28, 32], as well as syndromes contain CVM such as spondylocostal dysostosis in humans [33-35]. Disruption of WNT signaling also affects somitogenesis, whereas cyclic Axin2 expression is maintained when Notch signaling is impaired [36]. *Fgf8* is proposed to encode wavefront activity because experimental manipulation of *Fgf8* levels caused corresponding shifts in the position of the determination front in cultured chick and zebrafish embryos [37, 38]. *FGF4* and *FGF8* comprise the wavefront activity in the process of somitogenesis [39].

In the clock-wavefront model, the PSM progressively segmented into repetitive somites, which was driven by the action of Notch signal pathways. Segmentation clock and the wavefront are consisted of a series of related genes and signaling pathway products. The segmentation clock receives oscillatory expression of the segmentation *HAIY1* gene and a series of oscillators, with several genes expressed periodically representing the “clock” portion of model. The wavefront portion of the model corresponds to mRNA regulated FGF and WNT gradient. In the paraxial mesoderm, the formation of segmentation clock depends on both the periodic expression of related genes, and genes having clock topologies acting though negative feedback loop mechanisms [14]. For instance, in the Wnt pathway, *Wnt3a* stimulates the expression of downstream β-cat, which actives the expression of the downstream the scaffolding protein (AXIN2) and the soluble inhibitor Dickkopf-related protein 1(DKK1). Both the AXIN2 and DKK1 proteins are negative-feedback inhibitors of *β-cat*, forming a cyclical change [40, 41]. Similarly, *FGF4* and *FGF8* genes in the FGF pathway stimulate the expression of phosphorylated ERK (pERK) as well. This initiated the expression of downstream genes that encoded dual specificity protein phosphatase 4 (DUSP4), dual specificity protein phosphatase 6 (DUSP6) and Sprouty 2 (SPRY2), which are negative-feedback inhibitors of the FGF pathway [42]. In the Notch pathway, activation of the *Notch ligand Delta-like gene 1* (*DLL1*) results in expression of the downstream transcriptional effector Notch intracellular domain (NICD), thereby activing the expression of Notch targets *lunatic fringe* (*LFNG*) and Notch-regulated ankyrin repeat protein (NRARP), which are negative-feedback inhibitors to NICD [43, 44]. In addition, pERK and NICD have been also reported in the stimulation of *HES7* gene which is a negative-feedback inhibitor of pERK and NICD as well [45, 46].

**Figure 1:**
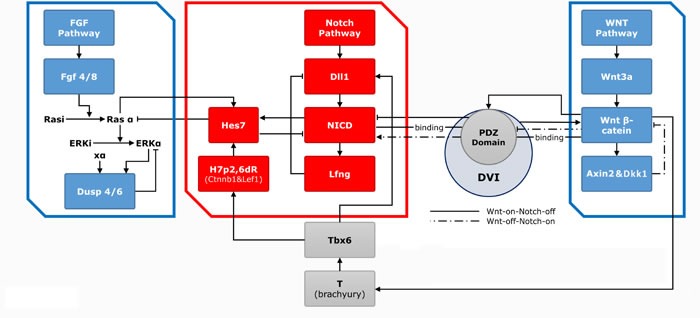
The three main pathways with related genes and effectors in the somitogenesis The clock (red frame)-wavefront (blue frame) model and components are shown. The clock is mainly consisted of Notch pathway and series of genes and effectors. *Dll1* and its downstream NICD and *Lfng* expressed periodically through a feed-back loop to form the “clock” model. FGF and WNT signal gradients constitute the wavefront. In the Wnt pathway, *Wnt3a* induces the expression of downstream *β-cat* and the *Axin2*. The AXIN2 and DKK1 proteins are both negative-feedback inhibitors of β-cat, thereby forming a cyclical change. *Wnt3a* induces the expression of *Tbx6*, which further activates the expression of *Dll1* and connects with NICD through *Hes7*, thus establishing communication rapport of Notch and WNT pathway. In the FGF pathway, *Fgf4* and *Ff8* genes induce the expression of pERK, which further prompts the expression of downstream *Dusp4* and *Dusp6*. The FGF signal oscillation is also based on a feed-back mechanism.

When the paraxial mesoderm cells receive a clock signal, *MESP2* is activated by NICD (Notch signalling) and *TBX6*. This action can be repressed by pERK. *MESP2* is initially expressed in a restricted area (one segment length) and subsequently with *Ripply1/2* expresses in the posterior half region, thus defining future segment boundaries [47]. The downstream target gene-*RIPPLY2* is activated, which is thought to be a negative-feedback inhibitor of *MESP2* and *TBX6*. The process contributes to the definition of the anterior boundary of the newly-formed segment [48]. Inactivation of *MESP1* and *MESP2* results in failure of paraxial mesoderm formation, indicating their importance in somitogenesis. The main components are summarized as follows (Figure 1) [14, 41, 49-53].

## THE IMPORTANCE OF TBX6 AND ITS RELATED GENES IN THE CLOCK-WAVEFRONT MODEL

It has been reported that the interaction between *TBX6* and the clock-wavefront model-related genes or *TBX6* itself will result in abnormal formation of somites, contributing to CVM [14, 41, 54].

In the first stage of PSM formation, the *Wnt3a*, FGF receptor 1(*Fgfr1*) and the T-box transcription factor brachyury(*T*) play key role during the epithelial-mesenchymal transition. Any mutants lacking of these factors would lead to an early arrest of trunk elongation at approximately the 8- to 12-somite stage [55-58]. Later, the mesogenin 1(*Msgn1*) plays a key role in the process of PSM maturation. As is mentioned in early papers, *T* is a target gene of WNT signaling [59, 60], which is essential for the early stage of mesoderm formation and migration [61], and acts its upstream gene *Tbx6* [62]. The *Msgn1* is a direct target of the synergism of WNT signaling and *Tbx6* [63]. The Msgn1 expression is detected only in a subset of mesodermal cells with the levels of Wnt3a messenger RNA are reduced in the tailbud of such embryos at E9.5-E11.5 [63, 64]. Using the *Tbx6*-knockout allele described previously [18], the expression of Msgn1 is detected strongly downregulated in Tbx6-null mutant embryos, which indicts that Tbx6 acts upstream of Msgn1 [63]. Msgn1 is described previously that it is essential for the specification and maturation of the paraxial mesoderm downstream of Wnt3a [65, 66]. The *Msgn1*−/− mutant was found to contribute to a kinked neural tube, lack of paraxial mesoderm and an expanded tail region. The *Msgn1*−/− and *Tbx6*−/− double mutants also lead to a severely kinked neural tube, loss of posterior somites, severely aberrant anterior somites, loss of paraxial mesoderm and an expanded tail region [67]. These findings suggest that Tbx6 is in a close association with Msgn1 during the somitogenesis, and any mutant of the two factors will contribute to severe CVM.

*Wnt3a* connects its downstream AXIN2 and DVI protein that plays a key role in vertebral symmetry [52]. The interaction between *WNT3a* and *TBX6* may affect the development of somites during embryogenesis. It has been reported that both *Wnt3a* and *Tbx6* mutants could lead to the formation of ectopic neural tubes concomitant with the absence of paraxial mesoderm or posterior somites [17, 68]. The *Wnt3a*, *Msgn1* and *Tbx6* mutant mice possessed a complete absence of paraxial mesoderm posterior to 6/7 somite, and abnormal morphogenesis of the 1-6/7 somites [67]. At the headfold stage, *Tbx6* expressed predominantly the wings of nascent mesoderm, and in a small portion of epiblast cells at the site of cell ingression where it overlapped with *Wnt3a*, indicating the close interaction during the somitogenesis. Of the three mutants, the *Tbx6*^−/−^ mutant was found to contribute to the most severe somite segmentation defect, with an aberrant anterior somites, an absence of posterior somites, and a slightly kinked neural tube as well as an expanded tail region. The *Wnt3a*^−/−^ mutant showed the deficiency of posterior somites and a reduced tail region. The *Wnt3a*^−/−^ and *Tbx6*^−/−^ double mutants also lead to the most severe phenotypes (somites segmentation defects). This includes severely kinked neural tube, no notochord, a reduced tail region as well as lacking most anterior somites, which were small and lacked the stereotypical organization.

*Dll1* is synergistically regulated by WNT signaling and Tbx6, and is essential for the activation of Notch gene homologue 1(*Notch1*) in the PSM [62, 69]. *Dll1* is a downstream target gene of *Tbx6* in the paraxial mesoderm [51]. Rib-vertebrae is a hypomorphic allele of the *Tbx6* gene [70], and *Dll1* expression is described previously reduced significantly in the PSM of rib-vertebrae mutant embryos [71]. Hofmann et al [72] identified that Dll1 expression in Tbx6-null mutant embryos was severely down-regulated on E8, and Dll1 transcripts were not detected on E8.5. Dll1 and Dll3, tow Notch ligands, are known to be involved in R-C (rostral and caudal) somite patterning [73, 74]. White et al [75] found that although *Dll1* expression is not detected in the tail mesoderm as normally of the *Tbx6*-null embryos, Dll1 expression is restore in the *Tbx6^tm1Pa^/Tbx6^tm1Pa^ Tg46/+* (a permanent transgenic line used for rescue) embryos. These findings suggest the close relationship between *Tbx6* and *Dll1* during somotogenesis, any mutant in the factors may lead to severe CVM.

*HES7* is a key gene in somitogenesis because of its connection with all the three major pathways [76]. By gradually creating smaller promoter reporters, the X-gal staining of the PSM was lost, confirming that 400 bp *Hes7* promoter region plays a key role in *Hes7* promoter activity [50]. This 400 bp region is strongly conserved in humans and comprises binding sites for remarkable transcription factors such as Lef1 (Wnt pathway) [77], Ets (FGF pathway) [78] and *Tbx6* [18]. Cooperation between *Tbx6* and the Wnt pathway was hypothesized to upregulate *Hes7*, while *Tbx6* alone did not. The findings suggest a closely connection between *Tbx6* and *Hes7* in the somitogenesis, and contribute to the occurrence of severe CVM.

**Figure 2:**
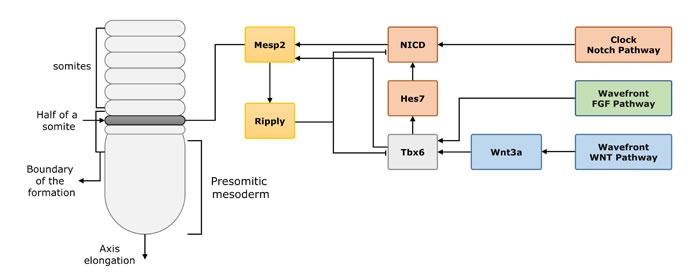
The process of vertebrate segmentation and the interaction of the related genes In the process of vertebrate segmentation, *Tbx6*, *Mesp2* and downstream *Ripply1/2* are important genes involved in somite segmentation. *Tbx6* stimulated by the Wnt and FGF pathway signaling, later induces the expression of NICD (also induced by Notch signal) and *Mesp2. Mesp2* initially expressed in a restricted area (one segment length), later with the *Ripply1/2*, (a feed-back inhibitor to *Tbx6* and *Mesp2*) expression in the posterior half region, which defines the future segment boundaries.

In the somitogenesis, somite segmentation is a key step to form the normal spine. *TBX6* and *MESP2* (activated by *TBX6)* and its downstream *RIPPLY2* are one of the pivotal factors in somite segmentation [14]. Takahashi et al [79] compared *Ripply1* null mutant, *Ripply2* null mutant and *Ripply1/2* double-null mutant mice with wild-type mice, to observe the region and quantity of *Tbx6*, *Mesp2* and *RipplyY1/2* expression during somite segmentation. In wild-type mice, *Mesp2* firstly expresses in a restricted area (a one-somite-length fashion), later with the *Ripply1/2* expression in the posterior half region. Under the interaction among the three factors, *Mesp2* expression is dependent on the *Tbx6* transcription factor and Notch signaling [80]. *Mesp2* leads to ubiquitin-dependent degradation of *Tbx6* proteins [81], and along with *Ripply1/2* contribute to degradation of *Tbx6* and *Mesp2*. Thus, *Mesp2* expresses in the anterior top of the *Tbx6* expression domain and form a next segmental border through the degradation of *Tbx6*. The *Tbx6* and *Mesp2* domain was expanded in *Ripply1* or *Ripply2* null mutant mice, while the posterior half region suffered segmentation defects. The segmentation defects become more severe in *Ripply1/2* double-null mutant. This process is summarized in Figure 2 [79, 81, 82].

## CLINICAL REPORTS OF TBX6 MUTATIONS CONTRIBUTING TO CVM

Several studies have reported that mutations in *TBX6*, such as single-nucleotide variation (SNV), may contribute to CVM (Table 1).

**Table 1:** Clinical reports of mutants of *TBX6* or *T* gene contribute to CVM

Authors	Year	Mutation types	Variants	Number of patients with mutations/total Number of patients	Vertebral Malformations	Other clinical manifestations
**Ghebranious et al.^[83]^**	2008	missense	T gene: c.1013C>T p.Ala338Val	3/50	Hemivertebrae, Butterfly vertebrae, Vertebrae fusion, C4 hypoplasia, Absent of S3-S5	Aortic stenosis, Bicuspid valve, Abnormalities of 1st and 2nd rib, Multiple left-sided rib fusions, Adducent left thumb, Conus terminates at L1
**Duncan B. Sparrow et al.^[84]^**	2013	stoploss	TBX6 gene: c.1311A>T p.^*^437Cysext	3/5	Scoliosis, Hemivertebrae, Fused vertebral blocks vertebral blocks	Short stature
**Shimojima et al.^[85]^**	2008	CNV	a 593-kb interstitial deletion of 16p11.2	2/3	Hemivertebrae of T10, T12, and L3^*^	A missing right twelfth rib, hypoplasia of the left twelfth rib
**Al-Kateb et al.^[86]^**	2014	CNV	deletion and duplication of 16p11.2 region	15	Congenital scoliosis, Idiopathic Scoliosis	Autism, spectrum, disorders, seizures, Behavioral abnormalities, developmental delay
**Qi Fei et al.^[87]^**	2010	SNP	rs2289292 and rs3809624	127	Congenital scoliosis	Deformities in syndrome associated with CVM
		CNV	deletion of 16p11.2 region + T-C-A#	17/237	Butterfly, Hemivertebrae, thoracic wedge vertebra	Rib abnormalities (missing ribs and bifurcation of ribs)
**Wu et al.^[88]^**	2015	frameshift	TBX6: c.1169_1170insC + T-C-A	1/237	Left L2 hemivertebra, T8 butterfly vertebra	None
		frameshift	TBX6: c.1250_1251insT + T-C-A	1/237	Left lumbar hemivertebra, atrial septal defect	Missing bilateral 12th ribs
		frameshift	TBX6: c.266_267insC + T-C-A	1/237	Left hemivertebra between T12 and L1	Missing right 12h rib
		frameshift	TBX6: c.704_705insG + T-C-A	1/237	Left T12 hemivertebra	Missing right 12th rib
		frameshift	TBX6: c.1179_1180delAG + T-C-A	1/237	Right T12 hemivertebra	Missing left 12th rib
		nonsense	TBX6: c.844C>T (p.R282X) + T-C-A	1/237	T1 and L3 butterfly vertebrae, Right T4 hemivertebra, Left T7 hemivertebra	None

CNV: copy number variation; SNP: Single nucleotide polymorphism

# T-C-A (rs2289292, rs3809624 and rs3809627) is risk haplotype in other allele of TBX6

* The proband and his mother share the same deletion, while his mother is a normal phenotype. The “Vertebral Malformations” and “Other clinical manifestations” refer to the proband's deformities.

Ghebranious et al [83] hypothesized that mutations in *T* and/or *TBX6* gene may lead to the occurrence of CVM. They reported fifty CVM persons, and sequenced the complete *T* and *TBX6* coding regions, splice junctions, and 500bp of the promoter region. Three unrelated patients harbored a same c.1013C > T substitution, resulting in a predicted Ala338Val missense alteration in exon 8 of the *T* gene while no variation was identified in*TBX6* sequence. Duncan B. Sparrow et al [84] used whole-exome sequencing to investigate a three-generation Macedonian family who is consisted of three SCD-phenotyped individuals. In the five members of the family, three of whom had clinical features and radiographic evidence of SCD, and two were clinically unaffected. The three affected individuals were confirmed to be heterozygous for the *TBX6* mutation, although unaffected individuals were homozygous wild-type indicating altered penetrance for the *TBX6* mutation. It is believed that the sequence variant disrupts the natural stop codon (c.1311A.T) and results in the addition of 81 nonsense amino acids to the C-terminus (p.*437Cext*81).

It has been reported single-nucleotide polymorphisms (SNPs) of *TBX6* were associated with CVM. Qi et al [87] genotyped two known SNPs in *TBX6* among 254 Chinese Han subjects(comprising of 127 congenital scoliosis patients and 127 controls). For the single SNP analysis, rs2289292 (SNP1, exon 8) and rs3809624 (SNP2, 5′ untranslated region) are significantly different between cases and controls (*P* = 0.017 and 0.033 respectively). The haplotypic analysis showed a significant association between SNP1/SNP2 and CS cases (*P* = 0.017), with the G-A haplotype more frequently observed in controls (odds ratio, 0.71; 95% confidence interval, 0.51-0.99).

Recently, there has been a focus on determining the relationship between copy number variations (CNVs) and CVM. Several articles reported CNVs in 16p11.2 region which harbors the *TBX6* gene may contribute to CVM. Shimojima et al [85] reported a 3-year-old boy with developmental delay, inguinal hernia, hemivertebrae of T10, T12, and L3, a missing right twelfth rib and hypoplasia of the left twelfth rib. The patient had a 593-kb interstitial deletion of 16p11.2 and the mother had the same deletion from the results of array comparative genome hybridization (aCGH). Al-Kateb et al [86] analyzed radiologic data obtained from 10 patients with 16p11.2 CNV (nine with deletions and one with duplication), and found that all the patients are affected with scoliosis, eight of them having congenital scoliosis (present at birth, a combination of scoliosis and vertebral deformities such as incomplete formation of vertebrae, failure of separation of vertebrae and a mixed one) and the remaining 2 having idiopathic scoliosis(cause unknown, sub-classified as infantile, juvenile, adolescent, or adult, according to when onset occurred, patients only appear as scoliosis). They additionally reviewed 5 patients reported previously with 16p11.2 rearrangement and similar skeletal abnormalities, concluding that two of them were affected with congenital scoliosis while the others had idiopathic scoliosis. Although there have been many reports on the association of 16p11.2 CNV with CVM, the exact mechanism was still unclear. Recently, Wu et al [88] elaborated the *TBX6* null variants and a *TBX6* common hypomorphic allele may contribute to CS with a compound inheritance model. They firstly enrolled 161 Han Chinese persons with sporadic congenital scoliosis, and 166 Han Chinese as controls. The CNV analysis identified 17 heterozygous *TBX6* null mutations in the 161 persons. This included 12 instances of a 16p11.2 deletion affecting *TBX6* and single-nucleotide variants (1 nonsense and 4 frame-shift mutations), and no such mutation was identified in the control group. Then they took analysis of 2 pedigrees, of which several unaffected family members showed a 16p11.2 deletion, and hypothesized that a heterozygous *TBX6* null mutation is insufficient to cause congenital scoliosis. They subsequently identified a common *TBX6* haplotype as the second risk allele in all 17 carriers of *TBX6* null mutations. The following replication studies in both congenital scoliosis cohorts and 16p11.2 CNV cohorts confirmed these findings.

## PROSPECT

CVM is a polygenic disease. In the somite development process, three major signaling pathways (Notch, FGF and Wnt pathway) make up the “segmentation clock - wavefront” model and many related genes are associated with *TBX6*. Therefore, mutations of *TBX6* and its interrelated genes may lead to the defects in somitogenesis resulting in CVM.

To date, the *TBX6* compound inheritance model shed new lights on the maximum ratio of CVM. The *TBX6* related mechanisms need further elucidation to provide more etiology explanation of CVM and potential targets for CVM prevention and intervention clinically.

Although clock-wavefront is the most popular model in studying somitogenesis, there are still other models. Further studies needed to be carried out to check whether TBX6 still plays a key role during somitogenesis, and how it works to cause CVM. Besides, it is confirmed that signaling network underlying the clock is markedly vary among species [89]. Researches for the mechanism of TBX6 lead to CVM are based on animal models at present. However, some animal models do not contain an exact *TBX6* homologous gene, such as zebra fish, which has *tbx6*, *tbx6l* and *tbx16* as the *TBX6* homologous genes [90]. More researches needed to be carried out to verify whether results from these animal models are the same in human.

Currently, the most accepted methods to identify genetic etiology of CVM are based on DNA analysis of SNP arrays or the next generation sequencing technology. However, there is no evidence whether DNA extracted from peripheral blood cells could restore the scene of vertebral development during somitogenesis. To achieve this, a much deeper research analyzing the deformed and normal tissues could be applied.

Actually, CVM is a complex disease which means several genes may involve in the pathogenesis. Indeed *TBX6* plays an important role during the somitogenesis, we cannot ignore roles of other genes and environment. For the further studies, we are also looking forward to see more findings revealed the roles of other genes and environment factors in the contribution to CVM.
